# The Winning Weaning Food (WWF): The Development of a Complementary Food for Food-Insecure Infants and Young Children in Malawi

**DOI:** 10.3390/nu11102292

**Published:** 2019-09-25

**Authors:** Rufus J. Theophilus, Markus Miller, Wilna H. Oldewage-Theron, John Dawson

**Affiliations:** 1 Department of Nutritional Sciences, Texas Tech University, Lubbock, TX 79409, USA; 2 Department of Animal and Food Sciences, Texas Tech University, Lubbock, TX 79409, USA

**Keywords:** food insecurity, malnutrition, infants, young children, complementary food, weaning, nutrients, Malawi

## Abstract

Growing evidence exists for the benefits of adequate infant and young children feeding (IYCF) practices at the weaning stage (≥ 6 months), including optimal growth, building the immune system, cognitive development, healthy food preferences, and reduced mortality and morbidity rates. However, these outcomes are not universally experienced. To ensure that a developing country such as Malawi, where recent studies have shown high rates of food insecurity and malnutrition benefits from adequate IYCF, five nutrient-dense complementary foods (Recipes 1 to 5) were developed. Standardized food processing techniques were used in the preparation and combination of Malawian indigenous food samples. The developed food recipes were assessed for nutrient density and cultural acceptability through sensory evaluations. Recipe 5 emerged as the winning weaning food (WWF), with an overall acceptability rate of 65% (mean score of 5.82 ± 0.87). Unlike theoretical analysis with the ESHA Food Processor, statistical analysis did not show that Recipe 5 met the Codex Alimentarius recommendations for macro- and micronutrients. However, it showed that the micronutrient recommendations for iron (*p* = 0.0001; 95%CI) and zinc (*p* = 1.00; 95%CI) were partially met, but not those for calcium and vitamins A and D. The prototype and outcome of this pilot study will be invaluable for interventions aimed at combating food insecurity and malnutrition in Malawi.

## 1. Introduction

Food insecurity continues to be a major global challenge confronting humanity and there are currently no adequate measures to address the situation. Meanwhile, food security should be every individual’s right and not merely a privilege. The right to food is enshrined in both international and national law. However, developed and developing nations still struggle with food sufficiency. For instance, despite the considerable progress that has been made in the past 25 years at increasing global food production, about 821 million people worldwide remain food-insecure according to a recent report on the State of Food Insecurity (SOFI) in the world [1]. This might be attributable to the fact that food insecurity is a complex phenomenon that affects food availability, affordability, the cultural norms that dictate acceptable means of acquiring food, and individual food utilization [2]. Furthermore, the FAO’s Africa Regional Overview of Food Security and Nutrition report of 2018 revealed that food insecurity rose from 20.8% to 23.2% for sub-Saharan Africa (SSA) between 2015 and 2017, making SSA the only region across the globe that has been consistently undernourished [3].

Malawi, as one of the developing countries in SSA, experiences severe food insecurity, leading to and/or resulting from malnutrition, with 37% of the population living below the poverty line [4]. Despite being known for cultivation of crops like maize and groundnut [5,6], the latest food insecurity response plan (FIRP) in Malawi shows that the emerging food insecurity situation continues to worsen, with 22% of the population experiencing severe hunger [7]. Moreover, the majority (45%) of Mzuzu households (the primary target population in Malawi) are severely food-insecure [8].

Although poverty/lack of income is the most prominent reason for this situation in Malawi, other factors contribute to food insecurity and malnutrition and have been documented [7,9]. The situation in Malawi signifies a grave danger to the food and nutrition security of not only infants/young children and their mothers, but entire households. Previous studies have linked household food insecurity with indicators of malnutrition such as wasting, underweight, stunting, and overweight/obesity [10,11,12]. Also, evidence of malnutrition exists across the globe: Over two billion people suffer from “hidden hunger” or deficiencies of micronutrients such as vitamin A, iodine (I), iron (Fe), and zinc (Zn), which are morphologically and physiologically essential [13,14]. For example, 63% of children in Malawi aged 6–59 months were reported to have iron deficiency anemia (<11.0 g Hb/dL), with 2% of children having severe anemia (< 7.0 g/dL). Also, one in three women aged 15–49 years is anemic [15]. Additionally, the 2018 FAO global report shows that 150.8 million and 50.5 million children under age five are stunted and wasted, respectively, with a greater percentage of this population living in developing countries such as in SSA [1]. 

Malnutrition is a major contributor to the global burden of disease, morbidity, and mortality among infants and young children, and a factor in about 53% of all childhood deaths worldwide. More than two-thirds of these deaths occur during the first 365 days and are often linked to improper feeding practices or inadequate complementary foods [16,17,18]. Malnutrition in infancy can be caused by inadequate breastfeeding or improper weaning—transitioning from breastfeeding/exclusive breastfeeding to complementary foods [19]. This is because at about six months and beyond, the demand for nutrient-dense food increases [20], and an inability to meet this demand often results in malnutrition. According to the Cost of Hunger study (COHA) of 2015, 23% of child mortality cases in Malawi are associated with malnutrition [21]. Moreover, only 15% of Malawian children below the age of five consume a minimum acceptable diet and 27% have a minimally diverse diet. Consequently, the combination of inadequate dietary diversity of Malawians (owing to overt dependence on maize), high disease burdens, poor sanitation and hygiene, and gender inequality contributes to undernutrition [7,15].

There is thus a need for a paradigm shift from the maize-dominated foods in Malawi to more diversified and nutrient-adequate foods that would address food insecurity and malnutrition as well as create job opportunities, boosting the economy and steering Malawi away from poverty to being a self-sufficient nation. A solution to the identified problems is proposed in this study through the focus on the development of diversified nutrient-dense complementary food recipes for IYC, made by combining locally sourced plant (e.g., soy) and animal (e.g., goat meat) foods and entrenching appropriate complementary feeding practices for sustainability.

In this pilot study, five nutrient-dense complementary food recipes were developed using the Codex Alimentarius guidelines on formulated complementary foods for older infants and young children, with the aim of choosing one, the winning weaning food (WWF), by sensory evaluation. 

## 2. Methodology

**Figure 1:**
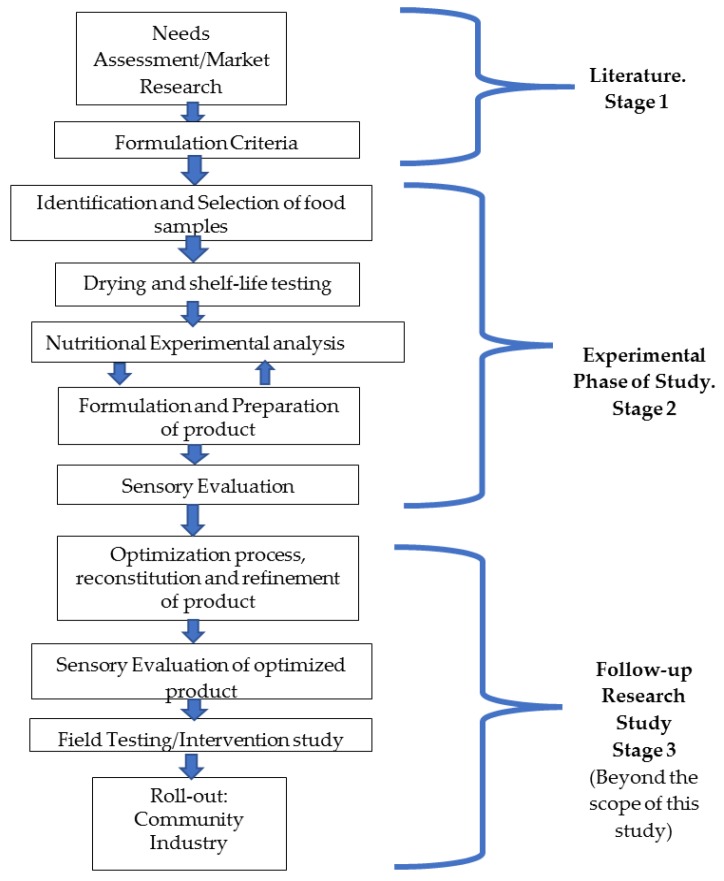
A schematic diagram showing the stages and processes involved in the development of complementary food (adapted from Zotor et al., 2015) [22].

The food processing approach deployed for the product development was a modification of Zotor and colleagues’ conceptual framework [22]. These stepwise approaches used in the development of the five nutrient-dense food recipes are summarized in the conceptual framework (Figure 1). The study design originally comprised three stages and 12 steps, as shown in Figure 1; however, only stage 1 and 2, with eight steps, were undertaken in this phase of the study. 

### 2.1. Stage 1

Step 1—Needs assessment/market research: Besides relying on the information provided by Malawian collaborators, a thorough desktop assessment was done to ascertain the food and nutritional challenges affecting infants and young children in Malawi. The literature search was done through Google Scholar, the Demographic and Health Survey (DHS) website for Malawian journal articles, Medline or PubMed, Cochrane Library, and Web of Science. Articles were searched using varying keywords like “infant,” “nutrition,” “infant food,” “infant weaning,” “complementary food,” “baby food,” “infant formula,” “infant feeding,” “infant nutrition,” “infant weaning formula,” “6–23 months children,” “introduction of complementary foods,” and “infant and young children feeding.” All articles related to the topic were considered, but priority was given to articles not more than five years old.

Step 2—Formulation of criteria: This was determined with the recommended specifications for supplementary foods in Malawi and the Codex Alimentarius standards [23]. The criteria included adequate nutrient density for complementary food measured per 100 g dry matter in terms of energy/macronutrients and micronutrients, affordability, cultural acceptability, and whether the food is safe for human consumption [8]. 

### 2.2. Stage 2

#### The Development of the Complementary Food

Laboratory assessments and experiments, chemical analysis of nutrient composition, meal preparations/cooking practical for formulated food recipes, and data capturing were done at this stage with raw food samples obtained from different grocery stores in Lubbock, TX, USA. 

Step 3—Selection of food items: Substitutes for Malawian indigenous foods were selected for analysis based on findings from the literature and reports from our correspondents. These staples were picked to provide adequate energy and make up for commonly observed micronutrient deficiencies such as vitamin A and D, iron (Fe), zinc (Zn), and calcium (Ca) [15], while considering their affordability and accessibility to the low-income population in Malawi. 

Step 4—Parboiling and roasting: Food samples such as kidney beans, butternut squash, and sweet potatoes were parboiled with a small amount of water to reduce nutrient loss and enhance taste and texture; soybeans and millet were roasted for similar reasons. Other food samples that did not require processing were directly subjected to drying.

**Figure 2:**
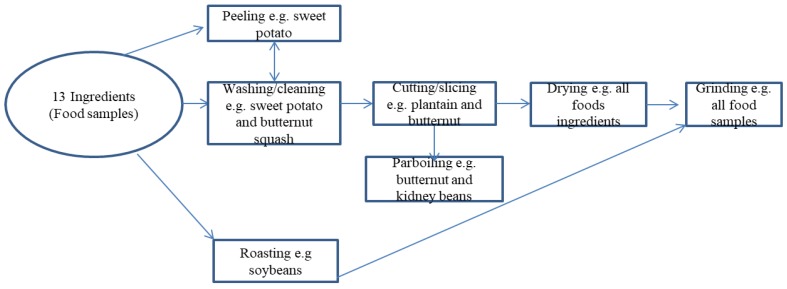
Food sample processing.

Step 5—Drying: A solar dehydrator, drum, and scientific ovens were used for drying in Malawi [24]. However, drying for this study was restricted to an electric/scientific oven. Food samples such as uncooked goat meats were treated with 3.5% salt and 3% vinegar/lemon juice, where appropriate, before being dried in an oven (as was done in Malawi). Butternut squash, plantains, and sweet potatoes were peeled, washed with water, cut open to remove the seeds (in the case of the butternut), and chopped into small pieces to enhance the drying process. Butternut squashes/pumpkins, sweet potatoes, and carrots were precooked before drying to avoid a raw taste and enhance the doneness. Most of the food samples (fruit and vegetables) were slowly dried at 65 °C to retain the quality of yield, while goat meat was dried at a higher temperature (127 °C) to prevent microbial activity. Food samples were typically dried within 24 to 48 h, ground, and temporarily stored in Ziploc bags at ambient temperature before analysis. Figure 2 describes the food processing approach adopted.

Step 6—Shelf life test: Dried food samples were tested for water activity (≤0.3) in order to gauge the microbial activity using a water activity meter. Carbonyl and thiobarbituric (TBAR) analyses were performed to assess the protein and lipid oxidation (rancidity), respectively, according to the modified Buege and Aust method [25], as described by Luque and co-authors [26]. 

Step 7—Nutritional experimental analysis: Food sample analyses complied with the standard protocols of the Association of Official Analytical Chemists (AOAC). Performed AOAC methods included: Moisture 950.46, ash 920.153, protein (automated method) 992.15, carbohydrates 986.25 (determination by difference) and advanced chloroform/methanol lipid extraction 982.23. A multianalyte method using flame atomic absorption spectrophotometry (FAAS) (AOAC 985.35) and the USDA wet ashing procedure were used to determine the micronutrients Ca, Fe, and Zn. However, the vitamin A and D values were obtained from the USDA database [27] owing to the inability of carrying out experiments after an HPLC machine breakdown. The resultant data from our lab were subsequently compared to the data obtained from the USDA, Kenya, and the West African food composition tables to observe possible similarities or differences. 

**Figure 3:**
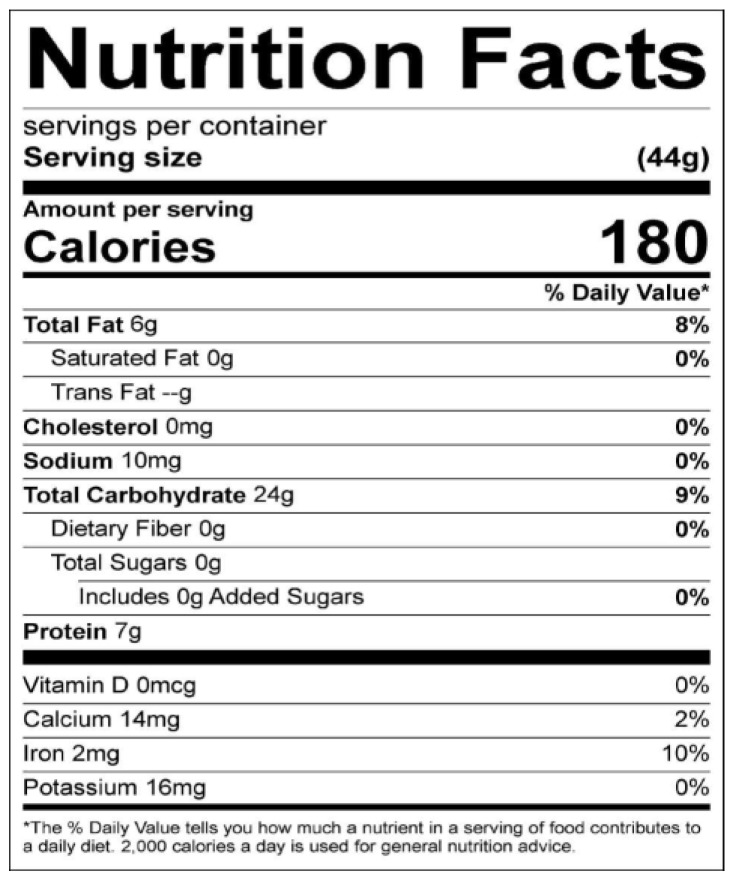
Food label for Recipe 5.

Step 8—Formulation and preparation of product: Formulations were based on the results of the nutrient composition of the analyzed food samples in accordance with Codex Alimentarius (CA) recommendations [28]. The results obtained for each category of nutrients were entered into the ESHA Food Processor as ingredients for 13 separate analyzed food samples and thereafter combined to form five different nutrient-dense recipes of complementary foods. The ESHA Food Processor was used to analyze the recipes and nutritional values obtained from total grams of each ingredient used (recipe quantifications) and generate corresponding food labels, as shown in Figure 3. The serving size specifications of the food labels aided in meal preparation. 

Step 9—Sensory analyses: A purposive sample of 50 panelists (58% male and 42% female) from Africa, but residing in the USA, was used to determine the sensory attributes of the developed recipe, after obtaining approval from the Institutional Review Board (IRB). The sensory session was conducted using a nine-point hedonic scale, with participants rating foods according to appearance, taste/flavor, texture/consistency, aroma/smell, overall acceptability, and most preferred recipe. Standardized methods, as outlined by the Society of Sensory Professionals, were used [29].

Data Analyses: All data analyses were performed with version 3.4.3 R programming language - Revolution Analytics (Dallas, TX, USA). A linear mixed model (LMM) was used to determine if there were significant differences between the food quality ratings as perceived by the sensory evaluators. The choice of LMM was because, as a consumer test, LMM mimics a “real-life” situation where panelists are treated as random effects. On the other hand, a one-sided (one-tailed) test was performed to ascertain whether the nutrient densities of the five developed complementary foods met the CA standards. 

## 3. Results and Discussion

Poverty, malnutrition, food insecurity, ignorance/inadequate nutritional knowledge, a lack of appropriate infant and young child feeding practices, a heavy burden of infectious diseases/illnesses, poor hygiene, and sanitation are major challenges confronting Malawians, as revealed by the needs assessment and market research phase of the study. Moreover, these factors are linked to other factors like drought, lack of water, and overdependence on social grants [30]. These factors, combined, are responsible for the high prevalence of maternal and childhood undernutrition, low life expectancy, and high infant mortality rate observed in Malawi [8,15,31]. 

Therefore, the solution to the identified problems was embedded in the formulation criteria for the various recipes. These criteria encompass: (i) nutrient density as recommended by CA. For instance, the nutrient density of the complementary food measured per 100 g (3.5 ounces) dry matter comprised a minimum of 410 kcal of energy or 4 kcal per gram weight on a dry basis with energy from protein being 15% maximum and at least 20% for fat. However, no stipulated amount was given for carbohydrates in the Codex document. According to the literature, about 45‒60% of energy per caloric serving or daily intake should be supplied from carbohydrates for children aged 6–36 months [32,33]. Notwithstanding, starch and fiber contribute to the total carbohydrates in food and are recommended by the CA [28]. Additionally, Ca, Fe, Zn, and vitamins A and D, which are essential for adequate growth, health, and cognitive development, were considered for the formulation criteria [34,35]. (ii) Developed recipes being affordable to the low-income population in Malawi when estimated by costing products in terms of dollars per kilogram (kg) for food ingredients using a nutrient calculator and/or the ESHA Food Processor. Moreover, the developed complementary food was cheaper than the commercial alternative (Lukuni kala), which costs $2.13 per kg from Mzuzu market. Although a previous study in Malawi documented the high cost of ASF, leading to its rare consumption [8], the present study addresses this issue. (iii) Cultural acceptability of the developed recipe, which was set at 60% for the sensory evaluation rating since this study served as a pilot study. (iv) Safety of food for human consumption, which was ascertained from a preliminary study involving the drying of food samples, a literature study, and a shelf life study. 

The outcome of the desktop assessment of nutritional needs, the formulation criteria, and information gathered from collaborators in Malawi informed the selection of 13 food samples, which included goat meat, butternut squash/pumpkin, kidney beans, cornmeal, sweet potatoes, spinach/pumpkin leaves, millet, soybeans, plantains/bananas, onions, carrots, mushrooms, and kale. Aside from goat meat, which was introduced in the developed recipes, all food samples are staples commonly consumed in Malawi and are suitable for complementary foods. Also, these food samples were selected based on their nutritional value and potential contribution to an optimized health status within the target population. 

Moreover, goat meat was selected as the source of heme iron in this study because it is one of the most common animals kept as livestock in Africa. Apart from being an age-old and high-quality source of meat and milk, goats have provided benefits to farmers and indigent dwellers in the developing world as they are adaptable to various climatic conditions, relatively cheap to rear, and their ability to survive diseases and feed effectively make them thrive on several natural resources that other livestock would not graze on [36]. In spite of being criticized for overgrazing, they can control bush encroachment and are a source of lean meat, which is nutritious and not taboo to people of different religions [37,38]. Also, goat-rearing ranks first in Malawi, above domesticated cattle, pigs, and sheep [39,40]. Moreover, a study based on trials with rats, which were fed 10% of protein from goat meat, showed that goat meat possesses a high biological value and a digestibility coefficient of approximately 60.4% and 97%, respectively [41,42], making it suitable for complementary foods.

The processing of the selected food samples (as in Figure 2) transformed the ingredients into flour for nutrient analyses and cooking. Notable changes occurred in the color and moisture content of food samples, as shown in Table 1 and Table 2, during the food processing stage. All food samples became flaky after the drying process, breaking/separating easily into tiny pieces when crushed. Table 2 shows the moisture content of dried food samples and raw food samples. The results achieved at least 60% moisture loss for raw food samples and less than the recommended 14‒15% moisture loss for dried samples [43,44]. According to Jay and colleagues, dried foods typically contain 25% moisture or less, with a water activity (a_w_) range of 0 to 0.60, while the moisture in shelf-stable foods is between 15% and 50%, with an a_w_ range of 0.60 to 0.85 [45,46], and they are said to contain an intermediate amount of moisture. The drying process significantly reduced the moisture content and extended the shelf life of food samples to at least six months.

The changes in color resulted from the effect of temperature and drying time on food sample. Aside from color, higher temperature and more drying time affect the taste and nutrient contents of products. The smallest observable change in color happened when the temperature was low (about 40 °C) or high (about 90 °C) with a shorter drying time [47].

Although microbial tests (for lipid and protein oxidation) to determine the shelf life of dried goat meat after keeping for six months appeared negative, such tests were not carried out on the remaining food samples as ample evidence exists in the literature of their shelf stability when optimally dried [48,49]. Furthermore, the results from the nutritional experimental analysis of the various food samples and the comparable values of vitamin A and D obtained from the USDA database are shown below.

As is evident from the results, most of the analyzed food samples such as onions (437 kcal), soybeans (455 kcal), millet (405 kcal), goat meat (393 kcal), kale (379 kcal), carrots (366 kcal), mushrooms (362 kcal), kidney beans (361 kcal), cornmeal (343 kcal), butternut squash (385 kcal), sweet potatoes (375 kcal), and plantains (377 kcal) are nutrient-dense food options, with spinach (329 kcal) being the least nutrient-dense. Nutrient density can be described as the nutrient content of foods expressed per 100 g (as in Table 3), or 100 kcal, or per serving size. It is often an estimation of the nutrient to calorie ratio. A clear distinction between nutrient and energy density is that while energy density solely describes macronutrients (carbohydrates, fats/lipids, and protein), nutrient density refers to nutrient-rich foods (NRFs) capable of supplying both macronutrients (e.g., protein and dietary fiber) and micronutrients (e.g., vitamins and minerals). According to Drewnowski and Fulgoni, another approach to visualizing nutrient density is calculating the percentage daily value (DV) of the various nutrients per serving of food, with respect to the total caloric content in the food. For example; a six-ounce plain (skim) milk yogurt that supplies < 5% DV of daily calorie but in turn supplies Ca > 30% DV, P > 25% DV, K > 10% DV, Zn > 10% DV and Mg > 5% DV can be said to be NRF owing to its useful nutrient-to-calorie ratio [50]. 

The carbohydrate contribution (per gram) in the different food samples is highest in plantains (89 g), followed by butternut squash (87 g), carrots with 84 g, sweet potatoes with 81 g, then millet with 77 g and cornmeal with 68 g. Other food items with higher (63 g) carbohydrates include kale, kidney beans, and onions. The protein contribution (per 100 g of goat meat) is 83 g according to this analysis. Two vegetables with higher values of protein obtained in this analysis were spinach (38 g) and mushrooms (37 g), which were closely followed by soybeans (35 g) and kale (27 g). The amount of fat per 100 g of food sample is highest in soybeans (19 g), followed by onions (18 g).

For micronutrients, the highest amount of iron was observed in millet (35 mg), followed by kale (13 mg), spinach (12 mg), goat meat (10 mg), and kidney beans (10 mg). Theoretically, the amount of iron in goat meat should supersede all other listed food items in this experiment, but studies have revealed the volatile nature of some micronutrients such as iron and vitamin C when exposed to heat [51,52,53]. The mineral ashing process, which was carried out under intense heat (550–600 °C), can be implicated in the relatively low iron content recorded for goat meat. Although other methods that do not interfere with the integrity of the mineral constituents of food samples exist, they were not deployed in this experiment. The amount of zinc (10 mg) was highest in soybeans, closely followed by mushrooms (8 mg) and kidney beans (8 mg). Calcium amounts also showed some variability in the analyzed food samples. For example, spinach (78 mg) was followed by soybeans (68 mg), kidney beans (42 mg), cornmeal (42 mg), kale (42 mg), and carrots (40 mg), while butternut (7 mg), sweet potatoes (9 mg), millet (5 mg), and plantains (1 mg) had low values. On the other hand, most of the analyzed food samples did not contain vitamin D, except for mushrooms with 7.0 mcg. High amounts of vitamin A were recorded in sweet potatoes (8512.2 mcg), carrots (3120 mcg), butternut squash (532 mcg), and spinach (500 mcg).

Since the analyzed food samples were obtained in Lubbock, TX, USA instead of Malawi (in Africa), it became necessary to determine the comparability of the nutrient compositions in order to verify the reliability of the nutritional experimental analysis results when formulating complementary foods for an African population. Moreover, a previous study has shown that variability in the nutrient composition of foods is inevitable with regional differences [54]. According to other studies, this variability may depend on plant variety, geographical conditions, or the methods of cultivation [55,56]. However, an attempt to compare all the analyzed food samples with nutrient information on other food composition tables from West Africa, Kenya, and the United States Department of Agriculture (USDA) was futile because of the differences in the states of some of the food samples; while all our results were derived from dried food samples, other nutrient composition tables were inconsistent, featuring results from fresh/wet food samples in some cases. An example is presented in Table 4, where the nutrient values for goat meat obtained from Lubbock could not be compared with those from West Africa, Kenya, and the USDA database as a result of the difference in state. 

Notwithstanding, Table 5 reveals how cornmeal or maize (which is a staple food in Malawi) fared when compared to results obtained from our in-lab analyses. Cornmeal makes a popular dish in Malawi known as “insima,” which is typically consumed as cooked porridge. Most of the nutrient values obtained from cornmeal samples from Lubbock compared favorably with all the other sources, except for a few nutrients with wide variations such as vitamin A and calcium. The reason for this disparity is the fortification of cornmeal in the USA. While other maize samples were not fortified with vitamin A and/or calcium, the sample obtained in Lubbock for the purpose of this analysis was generously fortified with vitamin A and calcium.

The ESHA Food Processor software was used for multiple combinations until a seemingly balanced recipe (in terms of the Codex recommendations for macro- and micronutrients) was obtained before generating a food label, as presented in Table 6. Each of the recipes (Recipes 1 to 5) was strategically developed to contain goat meat in order to ensure an adequate supply of protein and iron. Similarly, all five recipes contained sunflower oil. The introduction of oil became necessary owing to the difficulty encountered in meeting the macronutrient recommendations for fat (≥ 20% of total energy (TE)) during the formulation process. Attaining micronutrient adequacy for all of the developed recipes was difficult when theoretically and statistically analyzed using the ESHA food processor and the one-tailed test, respectively. Moreover, according to Osendarp and colleagues, infants and young children are often able to meet their macronutrient (e.g., protein) requirements by consuming locally available complementary foods, but not for “problem nutrients” such as calcium, iron, and zinc. They further revealed that optimizing the utilization of indigenous foods by increasing the frequency and amount of nutrient-dense foods will improve the intakes of these nutrients, but they still may only partially meet the iron and zinc requirements [57,58].

Additionally, a summary of the information on the food labels is presented in Table 7. The table gives a breakdown of the five developed recipes and some of their nutrient compositions, as generated by the ESHA Food Processor. All five recipes met the macronutrient codex recommendations for formulated complementary foods for older infants and young children. Although the recommendation for carbohydrates remains unclear in the Codex documentation, the prescription in the literature ranges from 45% to 60% when considering per caloric servings or daily intake [31,32]. Therefore, based on the recommendations in the literature, only Recipe 5 met the carbohydrate recommendations. Serving sizes for all recipes ranged from 44 g to 50 g, yielding a total energy range of 180 to 210 kcal. Aside from meeting the Codex recommendation of 4 kcal/g, this serving size is similar to Dewey’s recommendations for complementary food for infants in developing countries—about 200 kcal/day for infants of 6–8 months of age and 300 kcal/day for 9–11 months of age [59]. This implies that serving sizes for the developed recipe will increase as infants get older and the need to increase serving yields will be met by multiplying ingredients with desired factor(s) of 2, 3, or more. In other words, one recipe of about 44 g to 50 g (180 to 210 kcal) will be enough for one day for infants of 6–8 months of age. On the other hand, none of the recipes aside from Recipe 4 met the Codex micronutrient recommendation for iron; however, Recipe 5 was the closest to the recommendation at 4.5 mg/100 g (2 mg/44 g). No vitamin D was present in any of the recipes, including Recipe 5, which had mushrooms as one of the ingredients, as shown in Table 6. Analysis showed that despite the 7 mcg contained in the analyzed mushrooms, only 0.35 mcg/serving or 0.8 mcg/100 g was contributed to the recipe by mushrooms, hence the score of zero. Calcium was low in Recipes 1 to 5 compared to the 500 mg recommended by the Codex. Meanwhile, the amount of zinc was not reflected in the food labels by design of the ESHA Food Processor, but its contribution to Recipes 1 and 2 was 1.0 mg/serving (2 mg/100 g), 4 mg/100 g for Recipe 3, and 4.5 mg/100 g for Recipes 4 and 5. Therefore, Recipes 1 and 2 were close to the 2.4 mg/100 g recommended by the Codex, while Recipes 3, 4, and 5 met the Codex requirements (2.4–8.3 mg/100 g) for zinc. 

The sensory evaluation results showing the mean scores ± SD for each of the five parameters of appearance, taste/flavor, texture/consistency, aroma/smell, and overall acceptability are presented in Table 8. Although it was hypothesized that there would be no significant difference in the sensory evaluation results of the developed complementary foods, the results showed significantly different mean scores for appearance, taste/flavor, texture/consistency, aroma/smell, and overall acceptability across all the recipes evaluated. Recipe 3 had the highest mean ± SD score of 5.64 ± 0.54 followed by Recipe 5 with 5.62 ± 0.54 for appearance. However, Recipe 5 had the highest mean ± SD scores of 6.22 ± 0.51 and 5.8 ± 0.92 for texture and aroma, respectively. Recipe 3 had the highest mean ± SD score for taste (5.7 ± 1.0). 

**Figure 4:**
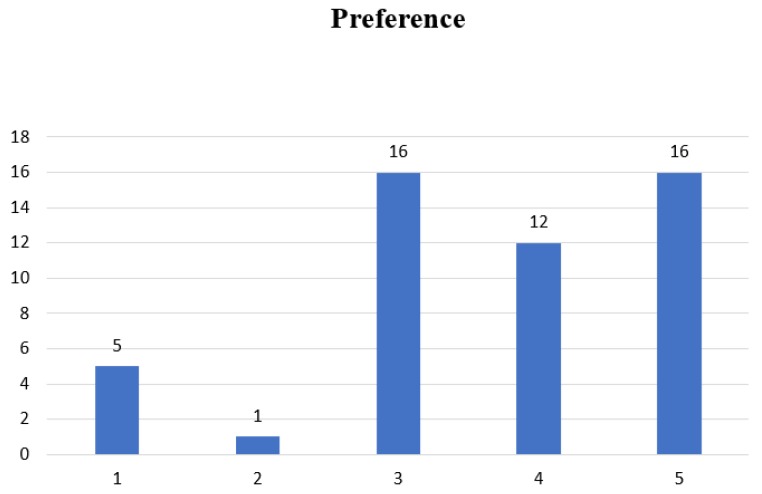
Bar chart showing the levels of preference for Recipes 1–5.

Additionally, the results in Figure 4 show that Recipes 3 and 5 are the preferred recipes (*n* = 16). Since both preference and overall acceptability tests were conducted simultaneously with the same tool, the highest mean score ± SD for the overall acceptability was 5.82 ± 0.87, in favor of Recipe 5. Therefore, a rank ordering of these five recipes based on the results of the sensory evaluation sessions shows that Recipe 5 (16) is the most preferred, followed very closely by Recipe 3 (16) and then Recipe 4 (12).

In order to statistically assess whether the nutrient densities of the five developed recipes met, exceeded, or fell below the Codex Alimentarius standards, a simulation was performed as there were no replicates of nutrient measurements in completed dishes but there were replicates of component nutrients. From the empirical component sample data, sample means and standard deviations for each component’s nutrients per standard unit (e.g., 1 g) were obtained. Then, for each recipe and nutrient, 10,000 simulations were performed as follows:

1. For each component used in the recipe, a nutrient value per 1 g was sampled from a normal distribution with mean and standard deviation as empirically estimated.
2. That sampled value was multiplied by the amount of the component used in the recipe.
3. An estimate of the total amount of the nutrient in a fully prepared dish was simulated by summing its components independently.

The above protocol provides an empirical reference distribution for the total amount of a given nutrient in a given recipe, which may then be used to determine whether recipe has met, exceeded, or fallen below the CA standard for that nutrient. For example, for a standard that we wish to meet or surpass, if half of the simulated values met or exceeded the standard, the corresponding *p*-value would be *p* = 0.5. In contrast, if none of the simulated values met or exceeded the standard, the corresponding *p*-value would be 1; and if all of the simulated values met or exceeded the standard, the corresponding *p*-value would be no more than *p* = 0.0001 (1 divided by the number of simulations). Note that these are one-sided tests, as it is desired to know whether or not the Codex Alimentarius standard has been met (one way or the other), not whether or not the amount of the nutrient in question is equal to the Codex Alimentarius standard. The simulation reference distributions may also be used to generate 95% confidence intervals for the average amount of each nutrient in each recipe. These are found in Table 9. 

The results from our simulations show that only Recipe 3 came close to meeting the Codex recommendation for protein. All recipes except Recipe 5 met the Codex standard for fat. Meanwhile, no specific recommendations for carbohydrates exist in the Codex document, but Recipe 5 achieved the range of 50‒55% that is recommended in the literature [30]. Similarly, the Codex micronutrient requirements were not significantly met except for iron with 12.13 mg in Recipe 5 and 6.97 mg in Recipe 4. Also, the amount of zinc (2.32 mg) in Recipe 5 came close to meeting the 2.4 mg Codex standard based on 5% bioavailability; however, it was not significant in this analysis. Based on the results obtained from this analysis (one-sided test), hypothesis 2, which proposed that the nutrient densities of the five developed complementary foods will meet or exceed/fall below (as appropriate) the standards in the Codex Alimentarius is not rejected. Although the one-sided *t*-test for the nutrient density of the developed recipes showed nutrient inadequacies in some cases, such deficits will be corrected during the optimization phase of this study, as described in the conceptual framework. Moreover, the challenges recorded in this study may have narrowed the chances of success. Such challenges may be associated with the identification and selection of food samples, the formulation approach, or the processing method adopted. For instance, despite conventional knowledge about fresh foods, all food items in this study were dried to extend the shelf life and prevent scarcity when they are out of season (in the case of fruit and vegetables). This invariably contributed to nutrient depletion, as seen in the results of this study. Discoveries from this study showed that homemade complementary foods can fill the nutrient deficits observed in both commercial complementary foods and those that are locally fabricated, provided that (1) challenges that contribute to nutrient loss during processing (e.g., drying) can be addressed; (2) fortificants bearing the required/deficient nutrients are added to the food as micronutrient powders (MNPs) and a small quantity of lipid-based nutrient supplements (sq-LNS) [58]. 

The strength of this study lies in its practicability. The researchers did not rely on the available food/nutrient composition tables, as is common in similar studies promoting food-based strategy of addressing malnutrition, but painstakingly analyzed all food samples considered in this experimental study. Although there have been several studies advocating the inclusion of animal-source foods (ASF) for adequate protein and/or micronutrient provision, this study is the first to create a prototype of complementary food for infants and young children using dried goat meat. Moreover, the collaborative nature of this study brought professionals from different fields of study together. For example, a team from a U.S. institution, comprising the Nutritional Sciences and the Food and Animal Science departments, and a team from a Malawian institution, partnered to ensure that the best practices were obtainable at every stage of this study. The use of the Codex Alimentarius guidelines for formulating complementary foods for infants and young children improved the quality of the five developed complementary foods and, by extension, counts as a strength of this study. Also, the use of the ESHA Food Processor constituted a major strength in that it allowed us to achieve results marked by accuracy, timeliness, and clarity of interpretation during the formulation of the developed complementary foods.

Although the study had many strengths, a number of limitations were also identified. First, the targeted population for this research study was Malawians. The effect of the geographical/regional differences in locations where this study was conducted and where the targeted populations are domiciled cannot be dismissed. For example, some food samples obtained in the USA are fortified, while that may not be the case among the studied population in Malawi. This contributed to the variability observed in the nutrient compositions of food samples. Second, the drying method in this study, involving the use of an electric oven, may not have yielded the best results because studies have shown that other methods involving extrusion or freeze-drying better preserve the flavor, color, texture, and nutritional values of food [46,60], while the methods we used might have contributed to nutrient loss and denaturing. Third, drying a limited number of food samples hindered the probability of success with a potential nutrient-dense food sample. In other words, drying more food samples will increase the chances of discovering more nutrient-rich foods. Fourth, the variability in the nutrient content of the various analyzed food samples shows that we cannot assume that the same nutrient contents will be present when Malawian ingredients are used. Fifth, the use of adult African sensory evaluators (consumer panelists) who are residing in the USA might have influenced the outcome of the sensory analysis because of possible distortions in taste preferences among panelists, as a result of exposure to other foods. However, further studies will be needed to substantiate this claim.

## 4. Conclusions

Irrespective of the inadequacies presented by the “problem nutrients,” this study has demonstrated the possibility of developing affordable, culturally acceptable, and nutrient-rich homemade complementary foods using locally sourced food materials such as vegetables combined with ASFs obtained by drying goat meat samples. However, aside from the proposed optimization of the WWF, to ensure an adequate dietary intake of calcium, iron, and zinc, it may be necessary to consume foods in their natural/fresh states if such options are available to avoid the nutrient depletion instigated by factors such processing, handling, or preservation. In addition, there is a need for an intervention incorporating nutrition education, to teach households about the appropriate dietary approaches and practices that will translate into effective behavioral changes, thereby optimizing the adequate use of locally available food options. Alternatively, other cost-effective approaches to achieving nutrient adequacy may include the fortification of foods and making supplements affordable and accessible to a low-income population. 

Although this food-based pilot study was targeted at households in low- and middle-income communities of developing countries like Malawi, the resultant therapeutic effects, as ascertained by a nutrient density assessment, will immeasurably benefit any private or corporate enterprise willing to commercialize the product among high-income communities as well. Therefore, Recipe 5, which was culturally accepted by 65% of the panelists, together with Recipe 3 with a 61% acceptance rate, will be introduced to Malawians for further intervention studies.

## Figures and Tables

**Table 1:** Moisture content of dried and raw food samples.

**Food Sample**	**Percentage Moisture Loss in Dried Foods**
Goat meat (dry)	2.64
Kidney beans (dry)	9.05
Millet (dry)	3.009
Soybeans (dry)	5.11
**Food Sample**	**Percentage Moisture Loss in Raw Foods**
Plantains (raw)	62.98
Sweet potatoes (raw)	82.11
Butternut squash (raw)	90.7
Kale (raw)	91.2
Spinach (raw)	92.4
Onions (raw)	88
Carrots (raw)	87
Mushrooms (raw)	90.5

**Table 2:** Color transformation after drying of food samples.

Food Ingredients	Color before Drying	Color after Drying
Goat meat	Dark red	Grayish brown
Butternut Squash	Yellow	Pale brown
Sweet potatoes	Red	Reddish brown
Spinach	Green	Dark green
Plantains	Yellow	Pale yellow
Red Onions	White flesh tinged with red	Dark brown
Carrots	Bright orange	Pale orange
Mushrooms	Grayish white	Dark brown
Kale	Green	Dark green

**Table 3:** Results obtained from chemical analysis of food samples sourced from Lubbock, TX, USA.

Food Sample (100 g)	Energy (Kcal)	Carbohydrate (g)	Protein (g)	Fat (g)	Iron (mg)	Zinc (mg)	Calcium (mg)	* Vitamin A (mcg)	* Vitamin D (mcg)
Goat meat	393.00	4.62	83.50	4.34	10.76	4.13	14.64	0	0
Butternut squash	385	87	6.42	1.19	0.28	0.82	7.68	532	0
Kidney beans	361.90	63.69	23.74	1.20	10.38	8.45	42.27	0	0
Cornmeal	343.35	68.32	7.07	4.50	5.10	6.04	42.45	121	0
Sweet potatoes	375.9	81.3	10.79	0.66	0.52	0.81	9.10	8512.2	0
Spinach	329	28.48	38.87	6.59	12.72	2.07	78.47	500	0
Millet	405.17	77.73	13.05	4.50	35.35	6.54	5.60	0	0
Soybeans	455.32	33.52	35.80	19.59	0.29	10.48	62.19	3.85	0
Plantains	377.2	89.1	3.67	0.53	0.39	0.82	0.72	45	0
Onions	473.35	63.29	13.67	18.19	2.17	4.04	18.17	1	0
Carrots	366.47	84.56	3.76	1.31	3.55	1.20	40.57	3120	0
Mushrooms	362.55	52.69	37.04	0.25	2.03	8.70	17.77	0	7
Kale	379.17	63.06	27.68	1.64	13.86	2.07	42.45	146	0

* Values from USDA database.

**Table 4:** Comparison of nutrients in dried goat meat for Lubbock, Africa, and the USDA.

Nutrient (Per 100 g Dried Goat Meat)	Own (Lubbock)	West Africa	Kenya	USDA
Energy (Kcal) kJ	(393) 1644	(216) 900	(192) 804	(143) 599
Protein (g)	83.50	25.40	26.90	27.10
Fat (g)	4.340	12.20	9.30	3.03
Carbohydrates (g)	4.62	0.00	0.00	0.00
Vitamin A (mcg)	0.00	0.00	21.00	0.00
Vitamin D (mcg)	0.00	0.00	0.00	0.00
Zinc (mg)	4.13	5.00	4.48	5.27
Calcium (mg)	14.64	16.00	14.00	17.00
Iron (mg)	10.76	3.30	2.60	3.73
Fiber	-	-	-	-
Ash	4.91	1.60	1.50	1.46
water (g)	2.64	56.80	57.10	68.21

**Table 5:** Comparison of nutrient values of dried corn: Lubbock, West Africa, Kenya, and the USDA database.

Nutrient (Per 100 g of Cornmeal)	Own (Lubbock)	West Africa	Kenya	USDA
Energy (Kcal) Kj	(343.35) 1436.57	(351) 1480	(345) 1450	(365) 1527
Protein (g)	7.07	9.70	7.94	9.42
Fat (g)	4.50	4.00	4.50	4.74
Carbohydrates (g)	68.32	64.5	63.40	74.26
Vitamin A (mcg)	121.00	0.00	0.00	0.00
Vitamin D (mcg)	0.00	0.00	0.00	0.00
Zinc (mg)	6.04	1.73	1.88	2.21
Calcium (mg)	42.45	18.00	24.00	7.00
Iron (mg)	5.10	3.80	2.60	2.67
Fiber	-	9.00	9.40	-
Ash	2.09	1.40	1.20	1.20
water (g)	18.02	11.50	13.60	10.37

**Table 6:** Food components of the developed recipes.

Recipe 1	Recipe 2	Recipe 3	Recipe 4	Recipe 5
* Goat meat	* Goat meat	* Goat meat	* Goat meat	* Goat meat
Sweet potatoes	Cornmeal	Plantains	Kidney beans	Mushrooms
Kale	Butternut squash	Onions	Spinach	Cornmeal
Sunflower oil	Sunflower oil	Soybeans	Millet	Carrots
		Sunflower oil	Sunflower oil	Sunflower oil

* Animal source of protein.

**Table 7:** Summary of the five developed recipes (food labels) as generated by the ESHA Food Processor.

Recipe	Serving Size/Container (g)	Total fat (g) (% TE)	Protein (g) (% TE)	Carbohydrate (g) (% TE)	Calories	Vitamin D (mcg)	Calcium (mg)	Iron (mg)	Zinc (mg)
1	50	16 (68)	8 (15)	8 (15)	210 (4.2 kcal/g)	0	7	2	0
2	48	15 (67)	7 (14)	9 (18)	200 (4.2 kcal/g)	0	7	1	1
3	50	13 (55)	8 (15)	15 (28)	210 (4.2 kcal/g)	0	11	1	2
4	44	10 (67)	7 (14)	9 (18)	210 (4.8 kcal/g)	0	11	7	2
5	44	6 (30)	7 (15)	24 (53)	180 (4.1 kcal/g)	0	14	2	2
Codex	100	≥ 20	6 to 15	-	400 (4 kcal/g)	5	500	5.8–10% bioavailability	4.1–10% bioavailability

Nutrient values are stated per serving size/container and not per 100 g, as in the last column for the Codex Alimentarius standard values. TE = total energy.

**Table 8:** Mean scores for sensory evaluation ratings.

Mean Scores (± SD)						Significance Difference between Recipes’
Parameters	Recipe 1	Recipe 2	Recipe 3	Recipe 4	Recipe 5	*p*-Value
Appearance	4.42 ± 0.54	5.22 ± 0.54	5.64 ± 0.54	4.72 ± 0.54	5.62 ± 0.54	3.00 × 10^−4^
Taste/Flavor	4.64 ± 1.0	3.14 ± 1.0	5.7 ± 1.0	4.9 ± 1.0	5.48 ± 1.0	1.10 × 10^−10^
Texture/Consistency	5.3 ± 0.51	5 ± 0.51	6.04 ± 0.51	5.46 ± 0.51	6.22 ± 0.51	9.60 × 10^−6^
Aroma	5.52 ± 0.92	3.44 ± 0.92	5.1 ± 0.92	4.94 ± 0.92	5.8 ± 0.92	1.80 × 10^−9^
Overall Acceptability	4.96 ± 0.87	3.44 ± 0.87	5.52 ± 0.87	5.14 ± 0.87	5.82 ± 0.87	2.60 × 10^−13^

**Table 9:** Nutrient densities of recipes compared with Codex recommendations.

Nutrient/ 100 g	Codex A	Recipe 1	Recipe 2	Recipe 3	Recipe 4	Recipe 5
Protein	6–15%	16.30 *p* = 1.00 95% CI: (16.2, 16.4)	16.54 *p* = 1.00 95% CI: (16.3, 16.8)	15.07 *p* = 0.52 95% CI: (12.7, 17.4)	19.91 *p* = 1.00 95% CI: (19.5, 20.3)	16.55 *p* = 1.00 95% CI: (16.5, 16.6)
Fat	20% minimum	33.85 *p* = 0.0001 95% CI: (33.7, 34.1)	31.44 *p* = 0.0001 95% CI: (30.9, 32.0)	26.37 *p* = 0.0001 95% CI: (26.4, 27.2)	24.06 *p* = 0.0001 95% CI: (23.4, 24.8)	14.25 *p* = 1.00 95% CI: (13.0, 15.5)
Carbohydrate	*N*	15.01 95% CI: (11.7, 18.3)	17.95 95% CI: (17.2, 18.7)	28.77 95% CI: (26.9, 30.6)	47.55 95% CI: (46.0, 49.1)	53.99 95% CI: (52.0, 55.9)
Zinc (mg)	(a) 8.3 (b) 4.1 (c) 2.4	0.53 *p* = 1.00 95% CI: (0.52, 0.55)	1.16 *p* = 1.00 95% CI: (1.1, 1.2)	1.98 *p* = 1.00 95% CI: (1.7, 2.2)	2.30 *p* = 1.00 95% CI: (2.2, 2.4)	2.32 *p* = 1.00 95% CI: (2.1, 2.5)
Calcium (mg)	500	6.66 *p* = 1.00 95% CI: (5.7, 7.6)	7.35 *p* = 1.00 95% CI: (6.6, 8.1)	10.53 *p* = 1.00 95% CI: (9.2, 11.9)	11.33 *p* = 1.00 95% CI: (11.1, 11.6)	14.18 *p* = 1.00 95% CI: (13.7, 14.7)
Iron (mg)	(a) 11.6 (b) 5.8 (c) 3.9	1.87 *p* = 1.00 95% CI: (1.3, 2.4)	5.71 *p* = 0.69 95% CI: (5.4, 6.1)	0.73 *p* = 1.00 95% CI: (0.65, 0.81)	6.97 *p* = 0.0001 95% CI: (6.7, 7.3)	12.13 *p* = 0.0001 95% CI: (11.9, 12.3)

(a) 15% bioavailability; (b) 10% bioavailability; (c) 5% bioavailability.
